# Bariatric Surgery and Liver Disease: General Considerations and Role of the Gut–Liver Axis

**DOI:** 10.3390/nu13082649

**Published:** 2021-07-30

**Authors:** Maria Cerreto, Francesco Santopaolo, Antonio Gasbarrini, Maurizio Pompili, Francesca Romana Ponziani

**Affiliations:** 1 Internal Medicine and Gastroenterology-Hepatology Unit, Fondazione Policlinico Universitario Agostino Gemelli IRCCS, 00168 Rome, Italy; mariacerreto@gmail.com (M.C.); santopaolofrancesco@gmail.com (F.S.); antonio.gasbarrini@unicatt.it (A.G.); maurizio.pompili@unicatt.it (M.P.); 2 Department of Translational Medicine and Surgery, Università Cattolica del Sacro Cuore, 00168 Rome, Italy

**Keywords:** NAFLD, MAFLD, bariatric surgery, gut microbiota, cirrhosis, liver transplant

## Abstract

Weight loss is a therapeutic solution for many metabolic disorders, such as obesity and its complications. Bariatric surgery aims to achieve lasting weight loss in all patients who have failed after multiple dietary attempts. Among its many benefits, it has been associated with the regression of non-alcoholic fatty liver disease (NAFLD), which is often associated with obesity, with evidence of substantial improvement in tissue inflammation and fibrosis. These benefits are mediated not only by weight loss, but also by favorable changes in systemic inflammation and in the composition of the gut microbiota. Changes in microbial metabolites such as short-chain fatty acids (SCFAs), capable of acting as endocrine mediators, and bile acids (BAs) as well as modifications of the gut-brain axis, are among the involved mechanisms. However, not all bariatric surgeries show beneficial effects on the liver; those leading to malabsorption can cause liver failure or a marked worsening of fibrosis and the development of cirrhosis. Nevertheless, there are still many unclear aspects, including the extent of the benefits and the magnitude of the risks of bariatric surgery in cirrhotic patients. In addition, the usefulness and the safety of these procedures in patients who are candidates to or who have undergone liver transplant need solid supporting evidence. This paper aims to review literature data on the use of bariatric surgery in the setting of chronic liver disease.

## 1. Introduction

Obesity has become one of the most frequent health problems in the developed world [1,2,3,4], being associated with a large number of comorbidities such as type 2 diabetes mellitus, hypertension, dyslipidemia, vascular disease, obstructive sleep apnea and several malignancies [1,5,6]. It has been stated that up to 75% of overweight patients and 90–95% of patients with morbid obesity have NAFLD [7,8,9], which represents a heterogeneous spectrum of liver alterations, ranging from simple fat accumulation (steatosis) to hepatic inflammation (non-alcoholic steatohepatitis, NASH), and even to the development of hepatic fibrosis/cirrhosis, and eventually hepatocellular carcinoma. NAFLD has an up to 30% reported worldwide prevalence [10,11,12,13] and carries a markedly increased risk of adverse outcomes and overall morbidity and mortality [14,15,16,17,18] (namely for cardiovascular and cancer-related diseases), having a very impressive impact on health care costs. NAFLD is often (36–67%) part of a wider cluster of metabolic abnormalities associated with obesity [10,19,20,21], referred to as “metabolic syndrome”.

## 2. Bariatric Surgery

The beginning of surgical procedures aimed at weight loss dates back to 1954 with the small bowel (jejuno-ileal) bypass procedure, followed by gastric stapling in the late 1960s and then, in the early 1990s, by laparoscopic surgery and gastric banding, while the use of endoscopic techniques is more recent. The mortality risk is related to the type of surgery performed, ranging between 0.1% for gastric banding and 1–2% for the other procedures [1].

The mainstay treatment of obesity relies on weight loss through lifestyle interventions including diet and exercise. However, these measures often do not lead to sufficient weight loss, and weight regain is common, failing to achieve a substantial and durable solution [22].

Another possible approach involves the use of medications, although their effectiveness seems to be limited, representing a short-term solution to a long-term problem [23]. The role of medications in obesity treatment is currently debated, due to two main reasons. Firstly, there is a poor weight loss and weight is quickly regained if the treatment is discontinuous. Secondly, there is no sufficient information on their long-term safety and their possible adverse effects. This is why the Food and Drug Administration (FDA) approved these drugs for no more than two years [23]. No long-term data are available for appetite suppressants, such as phentermine, benzphetamine, and phendimetrazine, which are currently approved for short periods, leading to an average weight loss of 3.5 kg [24]. Sibutramine is a norepinephrine and serotonin re-uptake inhibitor that leads to reduced food intake, but should be avoided in patients with cardiovascular disease, heart failure or arrhythmias [25]. Its efficacy has proven to significantly increase if used in association with lifestyle modifications [26]. Similar results have been accomplished for orlistat, which lowers energy absorption. It exerts a significant role in the amelioration of glucose tolerance and diabetes [27] and NAFLD improvement [28]. However, the mean weight loss after a treatment with orlistat is only 2.9 kg [29]. The decision to prescribe medications should be weighted according to the characteristics of the patient, because of their poor efficacy and the inconsistence of long-term data regarding safety. Compared with medical treatment, diet and physical activity should remain the favorite front-line approach to obesity.

Bariatric surgery is the only treatment with proven long-term weight control in obese adults [30,31], able to achieve a dramatic improvement or even complete resolution of comorbidities associated with obesity and to reduce long-term mortality [30,32,33].

However, studies have shown that various parameters can affect the good outcomes of bariatric surgery, with some patients experiencing insufficient weight loss (around 50%, with a difference between the procedures) or weight regain (around 20–25%)[34]. The mechanisms involved in these suboptimal responses are not completely understood, but the causes are probably multi-factorial [35]. They include sedentary lifestyle and low aerobic fitness [36], non-compliance to dietary programs (including grazing, sweet consumption, emotional eating, binge eating and maladaptive eating) [37], hormonal activity (for example, a reduction in the alteration of ghrelin, leptin, and incretins levels) [38], mental health causes and surgical procedure-related factors, such as the volume of the gastric pouch, gastro-gastric fistula or gastro-jejunostomy stoma dilatation [34]. A recent study outlined some preoperative factors related to weight regain after BS, emphasizing the importance of preoperative body mass index (BMI) and type of surgery [39].

The different surgeries produce effects on various mechanisms, including control of hunger, restriction of intake, change of appetite, diversion of food from the proximal part of the small intestine, malabsorption of macronutrients, food aversion, increased energy expenditure, and modifications of the gut microbiota and bile acid profiles.

The clinical indication towards bariatric surgery procedures is based on several considerations [4]: first of all, the presence of obesity, especially after various attempts to lose weight with non-surgical means; the potential risks and the commitment to attend the follow up program must also be taken into account. The choice of the surgical procedure rests on patient or surgeon preference, accessibility of appropriate aftercare, tolerance of risk, and permanent anatomical change. 

**Figure 1:**
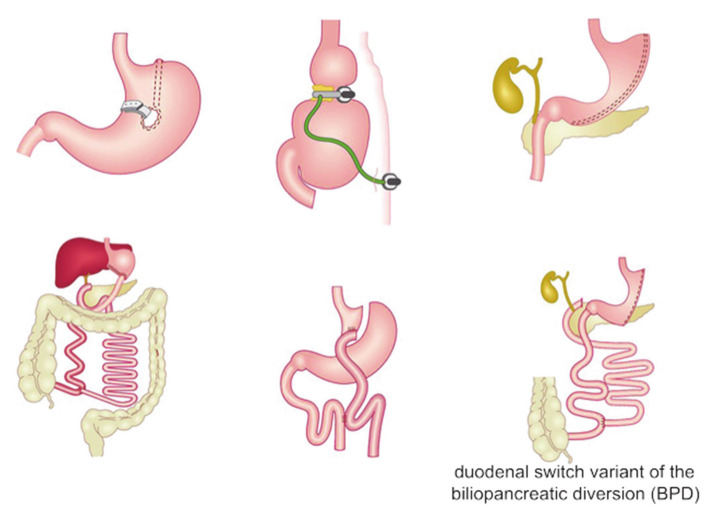
Description of the main types of bariatric surgery procedures. Figure was adapted from https://siceitalia.com/chirurgia-dellobesita accessed on 29 July 2021 (Italian Society of Endocrine Surgery—SICE).

Nowadays, bariatric surgery consists of three main kinds of procedures (Figure 1) [1,40,41,42], categorized in respect to their mechanism in:

restrictive procedures, aimed to decrease the stomach size in order to limit the intake of solids;

malabsorptive procedures, shortening the small intestine thus decreasing the surface area that is exposed to food and lowering the absorption of nutrients;

combined restrictive and malabsorptive procedures. 

### 2.1. Restrictive Procedures

Restrictive procedures include vertical banded gastroplasty, adjustable gastric banding (LAGB), sleeve gastrectomy (SG) and gastric imbrication. 

Vertical banded gastroplasty (stomach stapling), first described by Mason in 1982 [43], consists of banding and vertically stapling the upper stomach to create a small pouch along the inner curve. 

LAGB, first proposed in 1982 and widely spread with the advent of the laparoscopic technology in the early 1990s, is now considered one of the safest surgical options because of its effectiveness, minimal invasiveness and complete and easy reversibility. It involves inserting a gastric band around the cardia, within one centimeter of the esophago-gastric junction; this band is linked to an injection port in the subcutaneous layer of the anterior abdominal wall, thus allowing adjustability [44,45,46,47]. The optimally adjusted band exerts a pressure of between 25 and 35 mmHg on the gastric lumen, thus modifying the transit of the food bolus into the stomach and inducing a sense of satiety and a lack of appetite. LAGB, due to its burdensome aftercare needs, is contraindicated in those patients who cannot ensure a collaboration and cannot attend a regular follow-up.

SG is a non-adjustable and non-reversible laparoscopic procedure, which involves the creation of a narrow tube or sleeve by separating the lesser curve from the greater curve, thus exciding approximately 80% of the stomach by using a linear stapler so as to reduce food intake [1]. It has become popular for the so-called “sleeve and leave” approach: the surgery is easy, effective and does not require close follow-up. In addition, a series of hormonal changes occurring postoperatively (such as decreased levels of ghrelin and increased levels of peptide YY and Glucagon-like peptide-1) contribute to decreased appetite, reduced food intake and long-term weight loss [48,49,50]. Severe gastro-esophageal reflux disease can represent a contraindication for SG.

Gastric imbrication [1], also known as gastric plication, is a non-resectional variant of SG. The greater curve vascular pedicles are ligated, with the following imbrication of the gastric wall using two rows of sutures; this leads to the creation of a narrow lumen, similar in size to the sleeve gastrectomy.

### 2.2. Malabsorptive Procedures

Jejuno-ileal bypass (JIB) [1] is a malabsorptive procedure in which the proximal jejunum is diverted to the distal part of the gut, leaving a long portion of small intestine excluded with a strong reduction in absorptive capacity. It is able to achieve sustained weight loss and a great improvement in health benefits, particularly regarding lipid metabolism. However, due to its association with severe side-effects, such as electrolyte disorders, diarrhea, oxalate stones in the kidneys and progressive hepatic fibrosis with eventual liver failure (in which rapid weight loss, protein-caloric malnutrition, global malabsorption and endotoxin effects have all been implicated) [51,52,53,54], JIB has been progressively abandoned.

### 2.3. Combined Procedures

The biliopancreatic diversion (BPD) was first introduced by Nicola Scopinaro in 1976 [55], being subsequently abandoned in the last decade. It includes a restrictive component (a partial distal gastrectomy) and a malabsorptive component, consisting of the anastomosis of the distal side of a small intestine diversion (usually at 250 cm proximal to the ileocecal valve) to the gastric pouch and end-to-side anastomosis of the biliopancreatic limb to the terminal ileum (usually at 50 cm proximal to the junction with the cecum) [56].

In the duodenal switch variant of the BPD (BPDDS) [57,58], sleeve gastrectomy is performed so that pylorus and proximal duodenum can be preserved and anastomosed to the distal end of the transected small bowel, thus controlling stomach emptying and avoiding dumping syndrome. Through the bypassing of the proximal small intestine and the interruption of the enterohepatic circulation of bile, this procedure results in a great reduction of total cholesterol, triglycerides and low-density lipoprotein (LDL)-cholesterol. Insulin sensitivity is improved [59]. Malabsorption of amino acids can lead to hypoproteinemia, while the reduction of micronutrients and minerals intake can lead to osteoporosis and anemia [1].

Roux-en-Y gastric bypass (RYGB) is the combined procedure most frequently performed worldwide [60]. It was introduced in 1960 [61], and consists of a small upper gastric pouch, draining into a Roux-en-Y variable length limb of proximal jejunum (40 to 150 cm), with a distal excluded stomach. This technique has been repeatedly modified since its introduction, leading to the currently performed laparoscopic version, and typically results in 50–60% of excess weight loss (EWL) after 10 years. Different mechanisms play a relevant role in weight loss: the restrictive component of this procedure and the consequent slow emptying of the gastric pouch leads to an early satiation; the malabsorptive component, diverting the food away from the distal stomach, duodenum and jejunum, especially in the long-limb version of RYGB, reduces the digestion and the absorption of micro- and macronutrients; finally it can reduce sweet-eating thanks to dumping syndrome, which occurs after the intake of simple sugars or small osmotically-active molecules. 

Recently, an alternative form of gastric bypass has been introduced, known as single anastomosis gastric bypass (SAGB) or Mini gastric bypass [62,63,64,65], consisting of a loop of small bowel, a long and narrow lesser curve gastric pouch, and a longer bypass of the duodenum and proximal jejunum (usually 150 cm rather than the 40 cm limb obtained with Roux-en-Y).

SAGB proved to be simpler and safer than RYGB, though having similar results in improving metabolic syndrome and quality of life [66]. However, a large number of surgeons have strong objections to this procedure due to the risk of symptomatic (bile) reflux, marginal ulceration, severe malnutrition, and gastric and esophageal cancers [67].

## 3. Bariatric Surgery in Cirrhotic Patients

Surgical procedures in cirrhotic patients have been recognized to confer significant morbidity and mortality, thus causing a longer length of hospital stay and higher hospitalization charges [68]. Both the European (EASL) and American (AASL) association for the study of the liver guidelines consider portal hypertension to be a relative contraindication for surgery, based on clinical studies observing increased risk of postoperative liver failure, complications, and mortality in patients with portal hypertension compared to those without [69,70,71]. The Child–Pugh score, the model for end stage liver disease (MELD) and the presence of portal hypertension can be used to stratify the surgical risk [72,73,74,75]. In severe portal hypertension, preoperative portal decompression by Transjugular Intrahepatic Portosystemic Shunt (TIPS) placement has been considered as a possible solution in several small case series. TIPS has been used to treat portal hypertension complications such as bleeding varices, refractory ascites, hepatic hydrothorax and hepatorenal syndromes [76]. In a retrospective chart review of seven patients who underwent TIPS placement before elective abdominal or pelvic surgery, portal decompression has been proven useful in decreasing the risk of perioperative morbidity and mortality [77]. Similar results have been achieved by Azoulay et al. [78] and by Gil et al. [79], respectively, in seven and three patients undergoing major abdominal surgery. However, TIPS placement is not free of complications, and should only be performed by experienced interventional radiologists on selected patients [80]. 

There have been relatively few studies specifically addressing the complications of bariatric surgery in patients with cirrhosis (Table 1). Currently available data suggest that the less invasive laparoscopic procedures (RYGB, SG, adjustable gastric banding) have to be preferred [81,82], due to the low rate of serious complications, liver decompensation and postoperative mortality associated with them [83,84,85]. However, since the majority of the studies were based on patients with well-compensated cirrhosis, there is not yet an established consensus on which bariatric modality is best suited for the patient with severe, decompensated cirrhosis [86].

SG is commonly preferred for cirrhotic patients [82,83,84,87], because it reduces the risk of malabsorption (associated with RYGB) or placement of a foreign body (such as in gastric banding) and gives the possibility of a secondary access to stomach remnants and biliary system [90]. However, it may predispose to bleeding risk in the presence of gastric varices.

Ahmed et al. [91] recently published a review updating the 2015 systematic review by Jan et al. [92], which included 464 cirrhotic patients, 96.8% of them with well-compensated liver disease (Child–Pugh A). The 21 studies included in the review shared the conclusion that patients with obesity and liver cirrhosis represent a specific surgical challenge, due to increased morbidity and mortality risk from any intervention [81,91]; SG proves to be the best option for this category of patients, having significantly lower complication and mortality rates, and similar incidence of liver decompensation compared to RYGB [83,92]. Currently, SG and RYGB are the most common bariatric surgical procedures performed worldwide.

Nevertheless, more studies are needed to assess the long-term outcomes of patients with cirrhosis after bariatric surgery. Data on outcomes of bariatric surgery in patients with portal hypertension and Child–Pugh grade B cirrhosis are lacking; therefore, these patients remain poor candidates for bariatric surgery.

## 4. Bariatric Surgery and Liver Transplant

In liver transplant (LT) candidates, the choice of the type of bariatric technique is of major importance because of two factors: potential impairment of the absorption of immunosuppressive drugs and the chance of endoscopic access to the biliary tract. 

SG appears to be the most appropriate technique for this group of individuals, since it does not affect drug absorption and enables endoscopic access to the biliary tract in case of post-transplantation biliary structures [93]. In addition, SG has been shown to be safe and feasible in combination with LT both at a single setting and as a staged post-LT procedure [83,94,95]. 

The optimal timing to perform bariatric surgery in patients with cirrhosis who are candidates for LT is yet to be determined. Few studies including a small number of patients have been published so far (Table 2). Because of the higher morbidity and mortality observed among decompensated LT candidates, the pre-LT approach presents clear limitations. The concomitant performance of bariatric intervention and LT has the potential to minimize the number of surgical procedures in high-risk patients, but it requires simultaneous availability of both surgical teams and implies the risk of combining the complications of both procedures [96,97].

Based on this latter finding, although there is no consensus yet, the approach that is currently best accepted is to perform LT first and bariatric surgery later on [98,99].

## 5. Effects of Bariatric Surgery on the Liver

Since adipose tissue is a source of proinflammatory cytokines supporting the mechanisms involved in the development of NASH and fibrosis, favorable effects of bariatric surgery on liver function are expected to occur along with the progressive weight loss achieved in the long-term [6]. Meta-analyses confirm that histological features of NAFLD (steatosis, hepatocyte ballooning, lobular inflammation and fibrosis) improve or resolve in the majority of patients after bariatric surgery induced weight loss [100,101,102].

Taking into account the effect of specific procedures, LAGB results in a significant decrease of abnormal fattiness [103,104,105], liver volume and severity of steatosis [103,106,107,108], with an improvement in liver function tests [109,110], histology [111], serum adiponectin levels [109], glucose tolerance and insulin resistance. However, mild or moderate hepatitis has been reported [103,111]. 

SG leads to important improvements in aspartate aminotransferase (AST), alanine aminotransferase (ALT), triglycerides and high-density lipoprotein (HDL) serum levels [112,113], complete NAFLD resolution assessed with ultrasound imaging (especially in those who achieved more than 50% excess weight loss [114]) and histological amelioration [115].

An interesting case has been reported by Syed-Abdul et al. [116] on a 34-year-old woman who underwent sleeve gastrectomy. At 12 months after surgery she showed great improvements in BMI, liver enzymes level, NAFLD activity score (NAS) and fibrosis score. These significant improvements in liver histology and functionality were linked to the increase of hepatic fatty acid oxidation, representing the liver’s ability to burn fat. 

RYGB is associated with a reduction in steatosis, lobular inflammation, ballooning degeneration and centrilobular/perisinusoidal fibrosis [117,118,119,120]. These results were based on the comparison of a NAFLD activity score [121], NAFLD fibrosis score [122], liver enzymes [123] and liver biopsies taken before and after surgery [124,125]. 

However, literature data strongly support the evidence that the improvement in NAFLD occurs early after bariatric surgery, namely in the first 8–10 weeks, in a phase when no significant weight loss has yet been achieved [100,126,127]. Such a result may be probably related to the “acute” structural and endocrino-metabolic changes involved in amelioration of metabolic syndrome [106,128,129]. The causes of metabolic syndrome early improvement are complex, being probably connected with anatomical changes, such as duodenal exclusion and overflow of nutrients to the distal small bowel, and with the subsequent variations of the entero-insular axis mediated by gastrointestinal incretins (i.e., glucagon-like peptide 1 (GLP-1), and gastrointestinal insulinotropic polypeptide (GIP)) [130,131,132]. These hormones enhance adipokine metabolism, increase the production and release of insulin, and improve peripheral insulin sensitivity, thus favoring an anorexic state, and promote a modulation of the immune system with anti-inflammatory effects [106,130]. Post-surgical changes in the gut microbiota and bile acid circulation, as well as a decrease in portal influx of free fatty acids, may also be beneficial for metabolic syndrome and NAFLD, as suggested by recent studies [48,130,131,133]. As a particular example of early post-surgery modifications, LABG has been shown to induce a preferential mobilization of visceral fat, mainly in the first eight weeks [104]. Moreover, evidence suggests that BPD (with either Scopinaro’s technique or duodenal switch) is often characterized by a transient worsening of AST levels and hepatocellular necrosis in the first two–six months, promptly followed by a significant reduction of both these parameters and the improvement in histological features of NAFLD and NASH [134,135,136,137,138]. The rate of weight loss at two months, preoperative body weight, glucose levels and hepatic histology seemed to be useful in identifying patients at increased risk of acute liver damage, thus prompting the need for enhanced surveillance [134].

Concerning the post-surgery specific results on fibrosis and cirrhosis, literature data are not always consistent. Bariatric procedures leading to malabsorption may even result in the worsening of histology and sub-acute and chronic liver failure [8], probably due to the increase in pro-inflammatory cytokines at the time of surgery [136].

In 1973, Weismann et al. [139] first described one death due to liver failure in a series of 123 patients undergoing JIB. In the following years, many reports about progressive liver disease and acute liver failure after JIB were published [140,141]. There are two possible clinical presentations of liver damage: a rapid evolution to liver failure, usually within 24 months from surgery, or the development of a chronic liver disease evolving to cirrhosis. For example, a study including 453 patients who underwent JIB, with a mean follow-up of 12.3 years, reported a 10% prevalence of liver damage, including 24 cases of liver failure with 8 deaths, and 8% of cirrhosis [142]. There are multiple explanations for these negative effects on the liver. Every bariatric intervention implies rapid weight loss and lipid mobilization from peripheral deposits, leading to a massive release of free fatty acids (FFAs) that reach the liver, causing hepatotoxicity. However, malabsorptive surgeries such as JIB seem to have a particularly negative effect on liver function [143]. Different mechanisms have been taken into consideration: genetic determinants, severity of pre-operative steatosis, chronic protein malnutrition, toxic hepatic load of bile acids due to the disturbed enterohepatic circulation, bacterial overgrowth (caused by the presence of a blind loop with decreased motility) and portal endotoxemia can all contribute to the onset of liver injury and acute liver failure [144,145]. 

On the other hand, restrictive and combined procedures generally tend to induce a regression in nodules, fibrous bridging and cirrhosis grade [135,136,137,146], although some patients present no change or even a worsening in histological features [103,111,128]; this increase in fibrosis is probably associated with low–normal serum albumin, uncontrolled diarrhea, low intake of alcohol and menopausal status [136]. In less than one third of patients undergoing RYGB, steatosis and fibrosis remain unchanged after the intervention [147,148]; however, even if there are no modifications in the histological assessment, a marked decrease in the hepatic expression of different mediators involved in the regulation of fibrogenesis (collagen-alpha 1 (CO1A1), transforming growth factor-1 beta (TGF-1 beta), alpha-smooth muscle actin (α-SMA) and tissue inhibitor of metalloproteinase 1 (TIMP-1)), and inflammation (macrophage chemoattractant protein 1 (MCP-1) and interleukin 8 (IL-8)) has been reported [149].

Overall data suggest that RYGB is one of the most effective bariatric surgical procedures in terms of benefits on NAFLD, NASH and fibrosis, compared with SG and LAGB, probably due to the greater effect on weight-loss. However, SG determines a better improvement than RYGB in ALT, AST and GGT serum levels [113,150,151,152]. A recent study showed that RYGB and SG are equally effective for treating NAFLD and NASH, while RYGB has a better impact on hepatic fibrosis [153].

## 6. Effects of Bariatric Surgery on the Gut Microbiota

**Figure 2:**
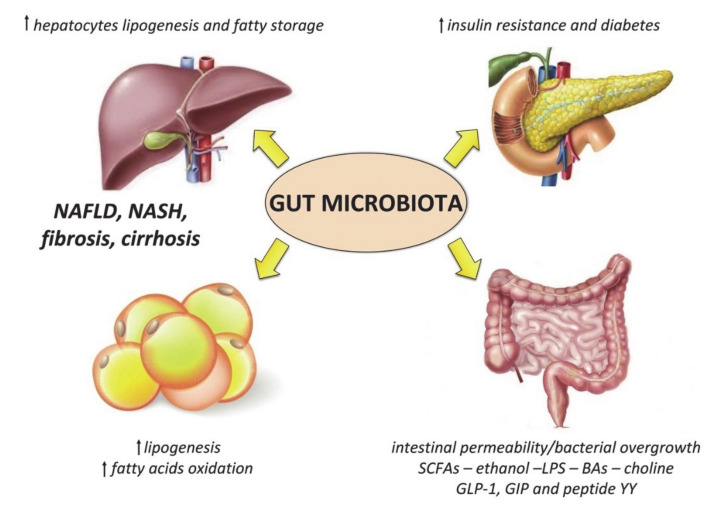
Mechanisms linking the gut microbiota to metabolic derangement in obese patients. Short chain fatty acids (SCFAs) derived from bacteria metabolism also influence lipid metabolism by increasing lipogenesis in the liver and in the adipose tissue, and inhibiting fatty acids oxidation; together with bile acids (BAs), SCFAs can modulate host appetite and insulin release through the secretion of gastrointestinal incretins (i.e., glucagon-like peptide 1 (GLP-1), and gastrointestinal insulinotropic polypeptide (GIP) and peptide YY), as well as the activation of the farnesoid X receptor (FXR). Choline depletion due to bacteria utilization also contributes to liver lipid accumulation, as well as the reduction of fasting-induced adipocyte factor (FIAF). LPS is another important modulator of glucose metabolism and insulin sensitivity. These alterations, associated with the increased intestinal permeability and bacterial overgrowth typical of obesity, as well as endogenous ethanol production by the gut microbiota, contribute to liver inflammation leading to non-alcoholic steatohepatitis (NASH) and fibrosis.

Specific changes of the gut microbiota composition occur in obese subjects, which mainly consist of: (1) reduced alpha diversity, (2) decrease in bacteria with potential anti-inflammatory properties (such as *Akkermansia muciniphila*) or other beneficial effects (such as *Lactobacilli* and *Bifidobacteria*), and (3) increase in pathogens (such as *Campylobacter* and *Shigella)* [154,155]. These alterations may contribute to fueling the systemic pro-inflammatory condition of these patients, as well as to the development and progression of metabolic dysregulation (Figure 2).

Many links have been discovered between several microbial-derived molecules and the pathogenesis of metabolic syndrome and obesity [156]. These molecules include lipopolysaccharide (LPS), a component of Gram-negative bacteria whose signaling cascades lead to the production of pro-inflammatory cytokines. LPS is associated with the development of obesity and increased insulin resistance, and obese patients have elevated plasma LPS levels [157,158]. Other small microbial molecules, including ethanol, trimethylamine (TMA), phenylacetate, and imidazole propionate, are also associated with metabolic dysregulation [156]. 

The gut microbiota is also involved in the pathogenesis of NAFLD and NASH with different mechanisms [159]: alterations of choline and bile acids metabolism, bacterial ethanol production, stimulation of hepatocytes lipogenesis and increased intestinal permeability [160,161,162,163]. Moreover, the interaction between bacterial antigens and cytosolic inflammasomes seems to accelerate the progression to NASH and hepatic fibrosis [164]. Spencer et al. [165] demonstrated that high levels of *Erysipelotrichia* and low levels of Gammaproteobacteria are correlated with a higher risk of developing NAFLD. Gram-negative bacteria, such as Bacteroidetes, appear to be enriched in NAFLD patients, supporting the role of LPS in metabolic dysregulation and inflammation [166]. Boursier et al. [167] showed the association between gut microbiota and disease aggravation from NAFLD to NASH; in particular, increased levels of Bacteroidetes were associated with NASH, while increased *Ruminococcus* with the severity of liver fibrosis.

### 6.1. Gut-Liver Axis and Liver Disease

Changes in the gut microbiota composition, metabolism and intestinal permeability affect liver structure and functionality. The explanation to this relays on the fact that about 70% of liver blood supply comes from the gut through the portal vein. Thus, in case the intestinal barrier permeability is increased, an interaction occurs between the liver and intestinal bacterial products or intact bacteria [168]. In fact, the translocation of gut bacteria, their products and fragments, from the intestinal lumen to the portal circulation, seems to correlate with NASH severity and its progression to fibrosis in obese patients [169]. NAFLD is also associated with increased intestinal permeability, involving both the epithelial and the vascular intestinal barrier [170,171]. Intestinal barrier impairment is a consequence of a variable combination of several factors, some of which precede and some of which follow the development of liver disease, such as: diet dysregulation (i.e., enriched in fructose or fat) [163,168,172,173], altered intestinal motility, bile acids enterohepatic circulation and gastric acid secretion, immune system dysfunction, intestinal congestion, and dysbiosis, even if the mechanisms involved in the interaction between microbes and the intestinal barrier are not completely cleared [174]. Translocation of gut-derived pathogen-associated molecular patterns (PAMPs) [175,176,177], such as LPS, activates pro-inflammatory pathways in the liver, leading to inflammation and development of fibrosis. In particular, LPS binds to the hepatic TLR-4/MD-2/CD-14 complex, which is over-expressed in obese patients, triggering MyD88-dependent or independent inflammatory pathways [169,176,177]. In addition, bacterial DNA and other bacterial components are involved in the activation of inflammasomes, thus improving the inflammatory response [173]. Gut dysbiosis is also associated with an excessive production of endogenous ethanol, which destroys tight junctions, increasing gut permeability and causing liver toxicity through direct or indirect mechanisms [178,179]. Gut bacteria are also involved in the development of NAFLD through the excessive storage of hepatic triglycerides by the acceleration of choline metabolism determining choline deficiency, which is responsible for the accumulation of triglycerides in the liver [169]. This can be exacerbated by the gut microbiota-mediated reduction of fasting-induced adipocyte factor (FIAF), an inhibitor of endothelial lipoprotein lipase (LPL). Moreover, there is an accumulation of hepatic free fatty acids due to the reduction in the activity of adenosine-monophosphate activated protein kinase (AMPK) caused by the excessive short-chain fatty acids (SCFAs). Finally, the gut microbiota can promote weight gain and liver steatosis, modulating host metabolism through the bile acid–farnesoid X receptor (FXR) signaling [180].

In conclusion, intestinal microbiome may have a relevant role in gut–liver axis homeostasis and in the pathogenesis of liver disease, especially NAFLD [167,176,177,181].

### 6.2. Bariatric Surgery and Microbiota

Bariatric surgery promotes evident changes in the gut microbiota composition, which are involved in weight loss and maintenance after surgery [182]. Literature data are consistent about the increased richness and evenness of intestinal microbiota after laparoscopic RYGB and SG [183]. These surgeries are associated with an increase in Verrucomicrobia (*Akkermansia muciniphila*), Proteobacteria (*Hemophilus, Rothia, Aggregatibacter, Citrobacter and Klebsiella*) and Gammaproteobacteria [182,184]. Both of these procedures lead to a decrease in the relative abundance of potential pathogens, such as *Escherichia coli* [185].

Bacteroidetes and Firmicutes are the two predominant phyla in the gut microbiota both before and after surgery [183]. They are also the most active groups of bacteria in individuals with severe obesity, with no significant differences between pre- and post-surgery [186]. Bariatric surgery can alter the Bacteroidetes/Firmicutes ratio, causing a reduction in Bacteroidetes and an increase in Firmicutes (especially *Roseburia*) [187,188,189]. However, this has not been confirmed in all human studies. Chen et al. [183] observed a decrease in Bacteroidetes after RYGB and SG, while there were no differences in Firmicutes abundance; however, 57.14% of the genera that were altered after surgery belonged to the phylum Firmicutes. Ilhan et al. [185] analyzed fecal samples of patients undergoing RYGB and noticed a difference between Firmicutes phylotypes. While *Streptococcus, Enterococcus, Lactococcus, Veillonella,* and *Granulicatella* were enriched, *Ruminococcus, Blautia,* and *Roseburia* were depleted after the surgery.

The gut microbiota composition after bariatric surgery can also influence the gut-brain axis and modify the inflammatory response and metabolism through various neural, hormonal and immunological pathways [190]. For example, the biosynthesis of a microbially-derived neuroactive intermediate, GABA (γ-amino butyric acid), is enhanced after RYGB, particularly due to the post-surgical abundance of *Enterococcus spp.* [191,192]. GABA has been reported to regulate gut motility and may affect food transition to the gut. Moreover, it stimulates the short chain fatty acids (SCFAs) butyrate, acetate, and propionate, a major class of bacterial metabolites obtained from fermentation of otherwise indigestible polysaccharides, have an important anti-inflammatory role in maintaining the intestinal barrier integrity [193], thus reducing the absorption of LPS. The consistent increase of SCFAs producers such as *Aggregatibacter,* Lachnospiraceae, *Rothia*, Ruminococcaceae, and *Streptococcus* after LSG and LRYGB is probably involved in the post-surgical attenuation of inflammation [189]. SCFAs also contribute to modulation of the host’s appetite and food intake, promoting the release of GLP-1 and peptide YY, by the interaction with G-coupled proteins expressed by enteroendocrine cells [194,195]. Moreover, SCFAs influence lipid metabolism through the increase of lipogenesis and the inhibition of fatty acid oxidation [153]. 

The composition of oral and fecal microbiota correlates with the weight-loss achieved after bariatric surgery, regardless of the type of surgical procedure [188]. Patients who achieved a %EWL of at least 50% six months after surgery had a predominance of Fusobacteria and Firmicutes in oral and intestinal microbiota, respectively [188]. Among patients who achieved less favorable outcomes, oral microbiota was enriched in Actinobacteria, while in the gut microbiota Bacteroidetes was the most prevalent phylum. In a cross-sectional study, patients who did not regain weight after RYGB had a higher alpha diversity, a greater abundance of Verrucomicrobia (*Akkermansia*) and *Phascolarctobacterium*, and a lower abundance of SMB53 compared with patients who regained weight [182]. *Phascolarctobacterium*, a SCFAs producer, correlated positively with weight loss; in contrast, studies suggest that the genus SMB53 contributes to obesity.

Weight-loss and alterations of the gut microbiota following LSG are associated with significant modifications in serum biomarkers [187]. Routine biochemical parameters such as cholesterol, low density lipoproteins (LDL), triglycerides, fasting blood sugar, aspartate aminotransferase (AST), alanine aminotransferase (ALT), blood urea nitrogen, creatinine and urea, glycated hemoglobin (HbA1c), the homeostasis model assessment insulin resistance (HOMA-IR), and serum concentrations of insulin, glucagon and inflammatory cytokines (IL-1β, IL-6, IFNγ), show significant decrease after surgery, while high density lipoproteins (HDL) and anti-inflammatory cytokines (IL-10 and TGF–β 1) increase [187,196].

Bariatric surgery, and especially RYGB, plays also an important role in bile acid (BAs) metabolism [197]. BAs have been shown to regulate glucose metabolism by increasing insulin sensitivity and reducing gluconeogenesis [130]; in particular, taurine-conjugated BAs promote GLP-1 secretion and energy balance by activating TGR5 [191]. In addition, BAs regulate the composition of microbial communities and the final outcome of surgical obesity in terms of body weight [158]. There is a double correlation between BAs and bacterial overgrowth; indeed, the lower concentration of BAs delivered to the colon due to entero-hepatic cycle diversion affects the composition of the gut microbiota, and the gut microbiota itself is involved in the modulation of BA metabolism [184]. Studies have shown that in NAFLD patients, a serum primary/secondary BA ratio is significantly higher compared to healthy subjects, and correlates with the severity of NAFLD [198]. Bariatric surgery can modify the intraluminal ileal environment, causing a significant repopulation of the gut microbiota and a reversal in the circulating primary/ secondary BAs ratio, thus inducing metabolic improvements with positive effects on NAFLD and metabolic syndrome [199]. In fact, the anatomical changes associated with RYGB create a shortcut for BAs to reach the lower intestine, thus allowing conjugated BAs to be actively reabsorbed in terminal ileum and primary BAs to enter the colon and be transformed to secondary BAs by the gut microbiota [154]. 

However, Seyfried et al. [191] conducted a study on the effects of RYGB on BAs levels in portal vein and peripheral plasma, demonstrating that caloric restriction had a more profound impact on the BA pools than RYGB. In fact, 3α-hydroxy-12 ketolithocholic acid, taurocheno-deoxycholic acid and tauro-β muricholic acid, were found to be higher in the portal vein blood compared to peripheral circulation in both RYGB and Sham-non-obese, but not in the Sham-obese group. Compared with the Sham-obese group, the RYGB group showed non-statistically significant lower levels of primary BAs (e.g., cholic acid (CA), chenodeoxycholic acid (CDCA), taurochenodeoxycholic acid (TCDCA) and α-muricholic acid) and secondary BAs (e.g., hyodeoxycholic acid, taurohyodeoxycholic acid and 3α-hydroxy-7 ketolithocholic acid) in the portal blood. 

A recent study conducted by Ilhan et al. [184] focused attention on fecal microbiota of obese patients who underwent RYGB; post-operatory changes appeared to be connected with a reduction in fecal BA concentration. They reported a drop in the quantity of primary (CA, taurodeoxycholic acid (TCA), glycocholic acid (GCA), and glycochenodeoxycholic acid (GCDCA), TCDCA) and secondary BA (taurodeoxycholic acid (TDCA), lithocholic acid (LCA), and glycolithocholic acid (GLCA)) both at 6 and 12 months after surgery.

The creation of a blind intestinal segment in RYGB is as always associated with small intestine bacterial overgrowth (SIBO), which is characterized by an excessive proliferation of bacteria in the small intestine [200]. These microbes include bacteria normally found in the colon, such as *Escherichia coli*, *Enterococcus* spp., *Klebsiella pneumoniae*, or *Proteus mirabilis*. The inflammatory response following the luminal overgrowth of atypical microbes leads to loss of integrity of the intestinal barrier, and to the synthesis of inflammatory cytokines which cause an impaired absorptive capacity of macro- and micro-nutrients. Moreover, the presence of bacteria with deconjugation properties is involved in fat and fat-soluble vitamin malabsorption, and in the production of LCA, which exerts potent toxic properties that exacerbate the intestinal epithelial cell dysfunction, and also contribute to carbohydrate and protein malabsorption [200].

Evidence indicates that RYGB-induced modifications of acid secretion and subsequent pH have an effect on the gut microbiota ecology in the stomach, too [197]. PH is markedly increased after RYGB, with an absence of both basal and peak-stimulated acid production in the small stomach pouch [201]. Some studies have demonstrated that pH modification affects genus and species relative proportions, and that a pH ≥ 4 enables bacterial overgrowth [202]. Moreover, the reduction of acid secretion has been linked with an increased number of Gram-positive bacteria and a decrease in lactic acid bacteria [154]; in addition, the modification of the total length of the small bowel after RYGB contributes to the growth of facultative anaerobes, i.e., Gammaproteobacteria [203].

As a final remark, gut mycobiota dysbiosis has recently been implicated in the pathogenesis of inflammatory and metabolic diseases. For example, an increased abundance of *Ascomycota*, *Saccharomycetes*, *Dipodascaceae* and *Saccharomycetaceae* has been observed in obese subjects [204]. Steinert et al. [205] noticed that, while bariatric surgery is associated with an increase in the gut microbiota alpha diversity, a similar change is not observed in the mycobiota. On the contrary, a significant reduction in mycobiota richness and diversity after RYGB was found, with clear but highly individualized changes in the fungal kingdom. Overall, *Ascomycota* and *Candida* were predominant before surgery in obese patients, thus supporting a link between these species and metabolic disorders and inflammation [204]; a decrease in *Candida* and *Saccharomyces* and an increase in *Pichia* was observed after surgery. However, RYGB surgery resulted in individualized changes of the fungal mycobiota, and not in unidirectional changes, as observed for bacterial microbiota [205]. 

## 7. Conclusions

In conclusion, the dramatic spread of obesity and metabolic syndrome, along with the associated liver diseases, raises the issue of finding a durable solution. Bariatric surgery currently represents the only treatment with proven long-term weight control in obese adults. It has shown promising results in improving metabolic disorders related to obesity, including NAFLD, NASH and hepatic fibrosis. The mechanisms by which bariatric surgery operates are not entirely understood, but they include control of hunger, increased energy expenditure, malabsorption of macronutrients, food aversion, and changes in the gut microbiota. In particular, its effects on the gut microbiota seems to have a great impact on weight loss and on the reduction of the pro-inflammatory conditions strictly connected with obesity, leading to a significant improvement in liver injury. It is not known exactly when the gut microbiota “turning point” occurs after bariatric surgery, so we can only analyze the effects of a mixed composition of changes. We believe that a model in which the individual parts are considered separately is no longer acceptable, given the dense network of metabolic connections in which the gut microbiota is involved.

The gut microbiota modulation represents one of the most promising research fields in the near future.

However, some major concerns regarding the research on the effects of bariatric surgery for treating NAFLD need to be emphasized. Firstly, most of the surgical studies are retrospective and not randomized or controlled, lowering the quality of the evidence.

Moreover, the outcomes are not always evaluated with standardized measurement, given that the methods employed variably include liver biopsy specimens, imaging studies, or other noninvasive methods. Histological assessment of liver biopsy should be the only parameter adopted to evaluate the presence of NAFLD and to quantify its possible regression; however, this procedure is not always proposed to the patient as it is not risk-free, thus performing it in individuals who do not require surgical interventions raises ethical considerations.

There is still insufficient high-quality evidence to recommend bariatric surgery for the treatment of NAFLD, hepatic fibrosis, or compensated cirrhosis; more data are needed to establish the best procedure to achieve satisfactory results even in patients with decompensated cirrhosis and in candidates for liver transplant. These should be the targets of future investigation.

## Figures and Tables

**Table 1:** Studies including cirrhotic patients with portal hypertension undergoing bariatric surgery.

Study	Study Design	N pts	Child–Pugh Score	MELD Score	Portal Hypertension	Type of Bariatric Surgery	Type of Associated Intervention	Efficacy	Factors Predictive of Efficacy	Safety	Factors Predictive of Safety
Kaul et al. [87] 2020	Retrospective review of prospective database	38 (22 cirrhosis; 16 stage 3 fibrosis)	A	-	1	20 cirrhosis 9 controls laparoscopic SG, 2 cirrhosis 4 controls RYGB, 3 controls OAGB	-	Average % EWL 65.8 ± 18.9%; Improvement on fibrosis stage	-	liver decompensation 1 (early) and 2 (late); 1 death	Previous portal hypertension
Quezada et al. [88] 2020	Retrospective, matched case-control	64 (16 cirrhosis; 48 controls)	A	7.4	3	11 cirrhosis 33 controls laparoscopic RYGB, 5 cirrhosis 15 controls laparoscopic SG	Liver biopsy	Average % EWL 84%	-	31% minor complications; 13% severe complications; 1 case of HCC after 6 months	Higher rate of complications in cirrhosis group
Salman et al. [89] 2020	Prospective case series	71 cirrhosis	A	-	26	SG	-	Average % EWL 21.7%; Fibrosis regression 67.7%	Amount of weight loss; sex	11 complications; 2 liver decompensations	Absence of end-stage liver disease, infections, good nutrition
Minambres et al. [83] 2019	Retrospective case series	41 cirrhosis	40 A, 1 B	7.2	11	28 SG, 11 RYGB, 2 BPD	-	Average % EWL at 5 years 21.16 ± 15.32%	-	7 complications; 5 liver decompensations	Age
Wolter et al. [85] 2016	Retrospective review of prospectively collected database	12 cirrhosis	-	-	-	150 SG, 146 laparoscopic RYGB, 6 BPD/GB	Liver biopsy	-	-	0.3% mortality; 16 major complications; 50 minor complications	-
Rebibo et al. [84] 2014	Retrospective, matched case-control	13 cirrhosis; 26 controls	A	7	-	SG	-	Average % EWL at 12 months 34.1% in the SG-cirrhosis group vs 33.1% in the SG group	-	No mortality or post-op complications	-
Shimizu et al. [82] 2012	Retrospective review of prospectively collected database	23 cirrhosis	22 A, 1 B	-	2	14 laparoscopic RYGB, l8 laparoscopic SG, 1 LAGB	2 laparoscopic SG with previous TIPS	Average % EWL at 37 months follow-up 67.7 ± 24.8%	-	No liver decompensation; complications in 8 patients	-
Dallal et al. [81] 2004	Retrospective case series	30 cirrhosis	A	-	-	Laparoscopic RYGB	Previous SG for 3 super-obese patients	Average % EWL 63 ± 15%	-	No perioperative deaths or liver failure; early complications in 9 patients; 1 unrelated death	-

Abbreviations: SG Sleeve Gastrectomy; RYGB Roux-en-Y Gastric Bypass; OAGB One Anastomosis Gastric Bypass; EWL Excess Weight Loss; LT Liver Transplant; HCC Hepatocellular Carcinoma; BPD Bilio-Pancreatic Diversion; GB Gastric Bypass; LAGB Laparoscopic Adjustable Gastric Banding; TIPS Transjugular Intrahepatic Portosystemic Shunt; MELD Model for End-stage Liver Disease.

**Table 2:** Studies including liver transplant (LT) candidates undergoing bariatric surgery.

Study	Study Design	LT pts (N)	Bariatric Surgery (N)	MELD Score at LT	Portal Hypertension	Type of Bariatric Surgery	Type of Associated Intervention	Efficacy	Safety	Factors Predictive of Safety
Fipps et al. [96] 2021	Retrospective cohort study	1416 pts who underwent LT for alcoholic liver disease	18	22 previous bariatric surgery 18 no bariatric surgery	/	16 RYGB; 2 laparoscopic GB	Following LT	5 years survival: 61.7% if previous bariatric surgery; 78.4% if no history of bariatric surgery	-	-
Pajecki et al. [98] 2014	Case report	-	1	31	1	Laparoscopic SG	Previous LT	EWL = 30 kg	No complications	-
Butte et al. [97] 2013	Case report	-	1	-	1	SG with Roux-en-Y biliary reconstruction	Intragastric balloon before LT	EWL = 46 kg. Normal liver function tests	No complications	-
Heimbach et al. [94] 2013	Prospective case series	44	7	19 LT 32 LT + SG	-	Previous weight loss + LT = 37 LT + SG = 7	-	Significant weight reduction	LT alone weight gain, complications, 3 deaths, 3 grafts losses	-
Lin et al. [95] 2012	Prospective case series	-	9	-	-	Laparoscopic SG = 8 SG = 1	Previous LT	Average % EWL = 55.5% No graft rejection	3 complications (mesh dehiscence, bile leak, dysphagia)	Previous surgical procedures and comorbidities

Abbreviations: LT Liver Transplant; MELD Model for End-stage Liver Disease; RYGB Roux-en-Y Gastric Bypass; GB Gastric Bypass; SG Sleeve Gastrectomy; EWL excess weight loss.
